# NRF2 Is a Potential Modulator of Hyperresistance to Arsenic Toxicity in Stem-Like Keratinocytes

**DOI:** 10.1155/2017/7417694

**Published:** 2017-09-10

**Authors:** Xiafang Wu, Bei Yang, Yuxin Hu, Ru Sun, Huihui Wang, Jingqi Fu, Yongyong Hou, Jingbo Pi, Yuanyuan Xu

**Affiliations:** ^1^Program of Environmental Toxicology, School of Public Health, China Medical University, Shenyang, Liaoning, China; ^2^Department of Histology and Embryology, College of Basic Medical Sciences, China Medical University, Shenyang, Liaoning, China; ^3^Experimental Teaching Center, School of Public Health, China Medical University, Shenyang, Liaoning, China

## Abstract

Arsenic is a well-known human carcinogen. Stem cells are indicated to be involved in arsenic carcinogenesis and have a survival selection advantage during arsenic exposure with underlying mechanisms undefined. In the present study, we demonstrated that CD34^high^-enriched cells derived from HaCaT human keratinocytes showed stem-like phenotypes. These cells were more resistant to arsenic toxicity and had higher arsenic efflux ability than their mature compartments. The master transcription factor in antioxidant defense, nuclear factor erythroid 2-related factor 2 (NRF2) with its downstream genes, was highly expressed in CD34^high^-enriched cells. Stable knockdown of *NRF2* abolished the hyperresistance to arsenic toxicity and holoclone-forming ability of CD34^high^-enriched cells. Our results suggest that skin epithelial stem/progenitor cells are more resistant to arsenic toxicity than mature cells, which is associated with the high innate expression of *NRF2* in skin epithelial stem/progenitor cells.

## 1. Introduction

Arsenic is naturally distributed in the environment as a component of water and soil and is also a contaminating metalloid dispersed by human activity [1]. Although arsenic has been identified as a human carcinogen with the skin as one of the targets [2], its carcinogenic mechanisms remain elusive. Transplacental carcinogenic activity of arsenic observed in humans [3, 4] and in experimental mouse models [5, 6] suggests that the fetal stem cell is a key target during carcinogenesis of this metalloid. Chronic exposure to arsenic has been shown to induce overabundance of stem cells or cancer stem cells during malignant transformation in various human cell lines [7–9], indicating a stem cell survival selection advantage in response to arsenic toxicity regardless of the tissue origin. With respect to the skin, *in vitro* models observed that short-term arsenic exposure maintained epidermal cells in a geminative state, blocking differentiation of the putative stem/progenitor cells [10]; chronic arsenic exposure induced an elevation in CD34-positive stem-like cancer cells during the process of malignant transformation of HaCaT cells [7]. *In vivo* study observed that in utero exposure to arsenite in mice led to disrupted homeostasis of skin stem cell [11] and increased risk of skin tumors with an accumulation of skin cancer stem cells as marked by CD34 [5]. However, no direct evidence for the effect of arsenic on skin stem cells is available.

Arsenic is a well-known oxidative stressor. As a master regulator of cellular antioxidant system [12], nuclear factor erythroid 2-related factor 2 (NRF2) is closely related to arsenic toxicity [13–16]. NRF2 regulates expression of downstream genes through the antioxidant response element (ARE) [17]. These downstream genes encode (1) antioxidant enzymes such as *γ*-glutamate cysteine ligase catalytic subunit (GCLC) and regulatory subunit (GCLM), (2) phase II drug-metabolizing enzymes such as heme oxygenase 1 (HMOX1) and NAD(P)H quinone oxidoreductase 1 (NQO1), and (3) multidrug-resistant proteins (MRPs) such as ATP-binding cassette subfamily (ABC) C1 (ABCC1), ABCC2, ABCC3, and ABCG2 [18–20]. Therefore, activation of NRF2-ARE pathway contributes to cellular defense and poses a survival advantage for cells in response to arsenic exposure [13, 16]. Supporting this theory, *Nrf2*-knockout mice are highly susceptible to oxidative damage or chemical carcinogenesis compared with the wild-type littermates [21, 22]. On the other hand, constitutive activation of NRF2 is suggested to result in a cancer phenotype [23], including in arsenic-induced malignant transformation of human keratinocytes [14], and moreover facilitate chemo- and radioresistance of cancer cells [24, 25].

Considering the observed overabundance of stem cells after arsenic exposure and the active role of NRF2 in chemical resistance [24–27], it is reasonable to assume that NRF2 is involved in stem cell biology and stem cell response to arsenic. Previous studies indicate that NRF2 regulates stem cell maintenance and differentiation; however, the reports are not consistent. For example, a reduction of high NRF2 activity induced cell differentiation and inhibited pluripotency in human embryonic stem cells (hESCs) [28], whereas in muscle and neural stem/progenitor cells, more evidence supported that *NRF2* was not highly expressed or activated until proliferation or differentiation occurred and therefore played roles in tissue regeneration [29, 30]. It appears that the function of NRF2 in homeostasis of stem cells depends on cell types and specific physical/pathological conditions. The role of NRF2 in stem cells, especially when they are under insult or stress, requires to be further elucidated.

CD34 is a surface marker for stem cells and cancer stem cells in the skin [31, 32]. In the present study, CD34^high^-enriched cells isolated from HaCaT human keratinocytes showed stem-like phenotypes and were used as a stem/progenitor cell model. Then, we tested our hypothesis that skin stem/progenitor cells possess a survival selection advantage in response to arsenic exposure. Furthermore, we found that NRF2 was more active in these stem/progenitor cells compared with the mature compartments and contributed to the hyperresistance to arsenic toxicity in these stem/progenitor cells.

## 2. Material and Methods

### 2.1. Cell Culture and Treatment

HaCaT human keratinocytes were obtained from the Shanghai Institute of Biochemistry and Cell Biology, Chinese Academy of Sciences (Shanghai, China). Cells were grown in DMEM medium (C11995500BT, GIBCO, Beijing, China) supplemented with 10% fetal bovine serum (FBS) (04-00101-A, Biological Industries, Cromwell, CT) and 1% pen-strep solution (03-031-1B, Biological Industries) under a humidified atmosphere of 5% CO_2_ and 95% air at 37°C. CD34^high^-enriched cells, CD34^low^-expressing cells, and passage-matched HaCaT (parent) cells were cultured on type IV collagen (3410-010-01, Trevigen, Gaithersburg, MD) and fibronectin-coated (610077, BD Biosciences, Bedford, MD) culture dish (or flask). Cells were maintained in EpiLife medium (MEPI500CA, Invitrogen, Shanghai, China) containing 5 ml of human keratinocyte growth supplement (S0015, Invitrogen) and 1% pen-strep solution. Cells at 80% confluence were exposed to sodium arsenite (S7400, Sigma, St. Louis, MO) as indicated.

### 2.2. Magnetic-Activated Cell Sorting (MACS)

MACS was performed with Mini MACS Starting Kit (130-042-301, Miltenyi, Auburn, CA) according to the instruction of the manufacturer. Cultured cells were trypsinized, resuspended in MACS buffer [calcium- and magnesium-free phosphate-buffered saline (PBS) containing 0.5% bovine serum albumin (BSA) and 2 mM ethylene diamine tetraacetic acid (EDTA)] at a concentration of 5 × 10^7^ cells/300 *μ*l, and incubated with FcR blocking reagent and CD34 microbeads (130-046-702, Miltenyi) for 30 min at 4°C. After washings, cells were passed through 30 *μ*M preseparation filter. CD34^high^-enriched cells were separated by positive selection on LS column (130-042-401, Miltenyi). Flow-through from positive selection was collected for CD34^low^-expressing cells.

### 2.3. Quantification of CD34^high^ Population with Flow Cytometry

Cells were detached by trypsinization, washed once with PBS, and counted. 5 × 10^5^ cells were suspended in 100 *μ*l MACS buffer, incubated with 20% phycoerythrin (PE) anti-human CD34 antibody (A07776, Beckman Coulter, France) or isotype control (A07796, Beckman Coulter) for 20 min at room temperature (RT) in dark, washed with 1 ml ice-cold MACS buffer, and centrifuged (300 ×g) for 5 min at 4°C. Cells resuspended in 0.5 ml PBS were determined using a Canto II flow cytometer (Becton-Dickinson, San Jose, CA) and analyzed by CellQuest software (Becton-Dickinson) or FlowJo (FlowJo, LLC).

### 2.4. Cell Viability

Cells were seeded in the 96-well plate at a density of 1.5 × 10^4^ cells per well and incubated at 37°C with 5% CO_2_ overnight. After treated with sodium arsenite at indicated concentrations for 24 h, cell viability was assayed using a CellTiter 96 AQueous One Solution Cell Proliferation Assay (MTS) Kit (Promega, Madison, WI) according to the manufacturer's instruction. This method measures the production of formazan catalyzed by mitochondrial dehydrogenase of viable cells. Formazan production was measured at a wavelength of 490 nm using a Synergy H1 microplate reader (Biotek, Vermont, USA).

### 2.5. Apoptosis Detected by Flow Cytometry

8 × 10^5^ cells per well were plated in the 6-well plate and allowed to attach overnight. Then, cells were treated with 20 *μ*M or 40 *μ*M sodium arsenite for 24 h, harvested with trypsinization, and centrifuged at 300 ×g for 5 min. Apoptosis was detected with TACS Annexin V Kit (4830-250-K, Trevigen) according to the manufacturer's protocol. Annexin V-FITC and propidium iodide- (PI-) staining cells were quantified with Canto II flow cytometer (Becton-Dickinson) equipped with CellQuest software (Becton-Dickinson).

### 2.6. Holoclone Formation

Holoclones are considered to be enriched in stem-like cells [33]. CD34^high^-enriched, CD34^low^-expressing, and parent cells were trypsinized, filtered with 70 *μ*m cell strainer, counted, and plated in the 6-well plate (2 × 10^3^ cells/well). The number of holoclones was counted under inverted microscope (DMI-LED, Leica, China) after 2 weeks.

### 2.7. Reverse Transcriptase Quantitative Polymerase Chain Reaction (RT-qPCR)

RT-qPCR was performed as previously described [9]. Briefly, total RNA was isolated using TRIzol reagent (Invitrogen, Carlsbad, CA), purified with RNeasy mini kit columns (Qiagen, Valencia, CA), and reverse transcribed to cDNA using Prime Script RT reagent kit (TaKaRa, Dalian, China). SYBR Premix Ex Taq Kit (TaKaRa) was used to assess cDNA amplifications. Data were analyzed using the delta-delta cycle time method. All primers were designed with Primer-BLAST online (http://www.ncbi.nlm.nih.gov/tools/primer-blast) and obtained from Sigma-Aldrich. Primer sequences are provided in supplemental material Table S1 available online at https://doi.org/10.1155/2017/7417694.

### 2.8. Western Blot

Protein isolation from whole-cell lysates was performed by using cell lysis buffer (Cell Signaling Technology, Danvers, MA) with 0.5% protease inhibitor cocktail (P8340, Sigma-Aldrich), 1% phosphatase inhibitor cocktail 2 (P5726, Sigma-Aldrich), and 1% phosphatase inhibitor cocktail 3 (P004, Sigma-Aldrich) on ice for 30 min. Protein extracts were separated by 7.5% SDS-polyacrylamide gel electrophoresis and transferred to polyvinylidene fluoride (PVDF) membranes (Millipore, Bedford, MA). The membranes were blocked with 5% nonfat milk and immunoblotted with primary antibody of p63 (20697-1-AP, Proteintech, Wuhan, China), OCT4 (11263-1-AP, Proteintech), keratin 5 (K5, Ab52635, Abcam, US), keratin 14 (K14, 10143-1-AP, Proteintech), SHH (20697-1-AP, Proteintech), NRF2 (sc-13032, Santa Cruz Biotechnology, Santa Cruz, CA), or *β*-actin (sc-69879, Santa Cruz Biotechnology) overnight at 4°C and with secondary antibodies (Santa Cruz Biotechnology) for 1 h at RT. Protein bands were detected with the Chemistar ECL Western Blotting Substrate (180-5001, Tanon, Shanghai, China), and the image was captured by 5500 Automatic Chemiluminescence Imaging System (Tanon).

### 2.9. Intracellular ROS

Intracellular ROS levels were evaluated by measuring dichlorofluorescein (DCF) fluorescence as described previously [34]. Briefly, cells were seeded in 12-well plates and treated with vehicle (Veh.), 20 *μ*M, or 40 *μ*M sodium arsenite for 2 h. At the end of the treatment, cells were incubated with 2 *μ*M 5-(and-6)-chloromethyl-2′,7′-dichlorodihydrofluorescein diacetate and acetyl ester (CM-H2DCFDA) (Molecular Probes, Eugene, OR) for 30 min at 37°C. The fluorescence was analyzed with Canto II flow cytometer (*λ*exc = 488 nm, *λ*em = 525 nm) (Becton-Dickinson).

### 2.10. Cellular Arsenic Accumulation and Export

Determination of cellular arsenic accumulation and export was modified from a previous report [35]. Cells were grown in arsenic-free medium to approximately 70% confluence. The medium was replaced with either fresh medium or medium containing 10 *μ*M sodium arsenite. After 24 h, to determine arsenic accumulation, cells were washed three times with PBS, harvested with trypsin, counted, and digested overnight with 50% perchloric acid : nitric acid (2 : 1) at 70°C; to determine arsenic export, PBS-washed cells were incubated in fresh arsenic-free medium for another 24 h. Then, medium was collected, and cells were trypsinized and counted. Arsenic concentration in the cell or medium was determined by graphite furnace atomic absorption spectrophotometry using a Perkin Elmer AAnalyst 100. Arsenic concentrations were normalized to cell number.

### 2.11. Silencing *NRF2* by Lentiviral-Based shRNA Transduction

MISSION shRNA lentiviral particles were purchased from Sigma-Aldrich. Transduction of HaCaT cells with lentiviral-based shRNAs targeting *NRF2* (SHVRS-NM_006164) or scrambled nontarget negative control (SHC002V) was performed as previously described [36]. Transducted cells were maintained in DMEM medium containing 1.0 *μ*g/ml puromycin (Invitrogen).

### 2.12. Statistical Analysis

Data were analyzed with GraphPad Prism 5 (GraphPad Software, San Diego, CA) and presented as mean ± standard deviation (SD). Unpaired Student's *t*-tests were used to compare the difference between Veh. and arsenic-exposed cells, as well as between scramble and *NRF2*-KD cells. Bonferroni posttests after one-way or two-way analysis of variance (ANOVA) were applied to other comparisons when it was appropriate. *p* < 0.05 was considered significant. All the experiments were carried out at least in triplicate.

## 3. Results

### 3.1. Survival Potential of CD34^high^ Subpopulation in HaCaT Cells in Response to Arsenic Exposure

**Figure 1:**
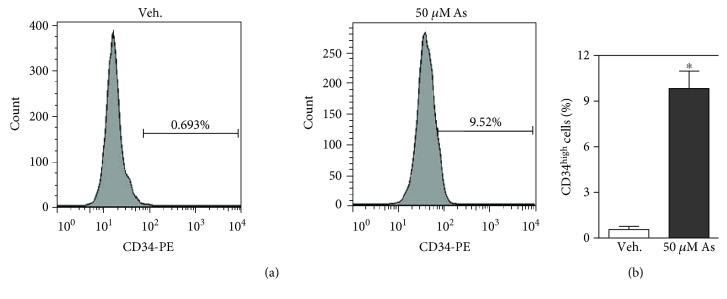
Flow cytometric analysis of the ratio of CD34-high-expressing (CD34^high^) cells in HaCaT cells. (a) Representative histogram of flow cytometric analysis for CD34^high^ cells. Left: HaCaT cells were exposed to vehicle (Veh., same volume of PBS to arsenic solution); right: HaCaT cells were exposed to 50 *μ*M sodium arsenite for 24 h. (b) Quantification of CD34^high^ population in HaCaT cells exposed to Veh. and arsenic. 1 × 10^5^ cells were counted for each sample. *n* = 3. ^∗^*p* < 0.05 compared with Veh.

CD34 is a surface marker for stem cells in the skin [31, 32]. We first assessed the presence of the cells with high expression of CD34 surface marker in HaCaT cells with flow cytometry (Figure 1(a)). CD34^high^ subpopulation is quite a small fraction, approximately 0.2–0.7% in HaCaT cells. After arsenic challenge (50 *μ*M, 24 h), CD34^high^ subpopulation was markedly increased to 8.9–11.1% (Figure 1(b)). Stem cells are resistant to chemical insult and are suggested to act as a “strategic” adaption in arsenic carcinogenesis [27]. One possible explanation for the observed increase of CD34^high^ subpopulation is the hyperresistance of CD34^high^ cells to arsenic-induced cytotoxicity. We tested this idea in the following study.

### 3.2. Stem-Like Characteristics of CD34^high^-Enriched Cells

**Figure 2:**
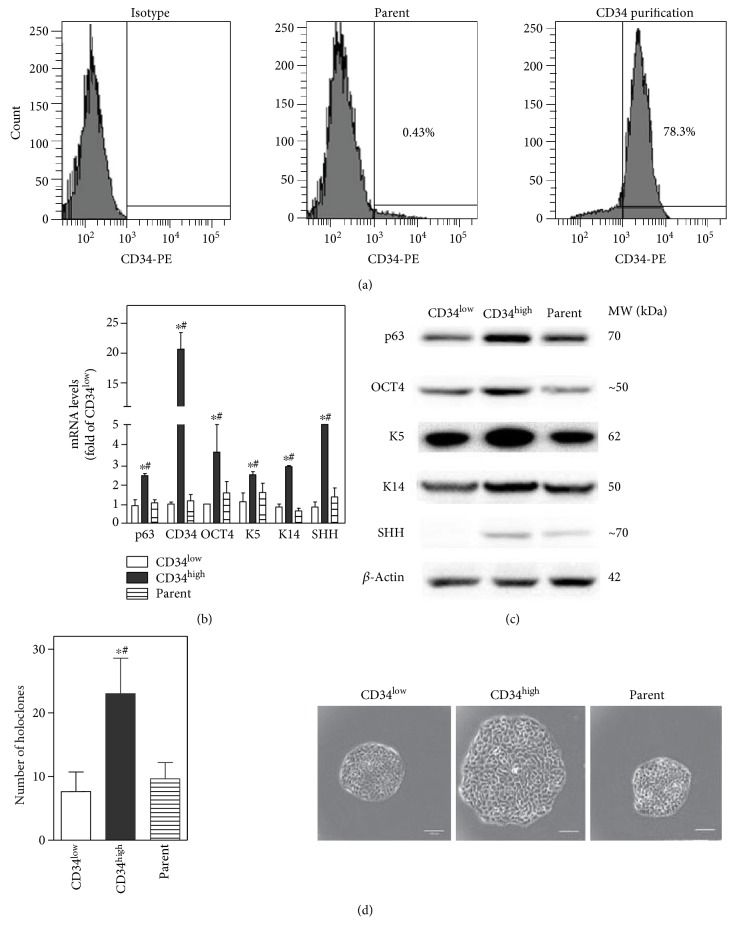
Stem-like characteristics of CD34^high^-enriched cells. (a) Flow cytometric analysis of the ratio of CD34^high^ cells. Left: isotope control of CD34 antibody; middle: the ratio of CD34^high^ cells in the HaCaT (parent) cells; right: the ratio of CD34^high^ cells after positive selection by CD34 magnetic beads. (b) mRNA and (c) protein levels of typical markers for undifferentiated epithelial cells or stem cells in CD34^low^-expressing, CD34^high^-enriched, and HaCaT (parent) cells. (d) Holoclone formation by CD34^low^-expressing, CD34^high^-enriched, and parent cells. Left: the number of holoclones was increased in CD34^high^-enriched cells compared with the CD34^low^-expressing or parent cells; right: representative image of holoclones (400x magnification, bar = 100 *μ*m). *n* = 3. ^∗^*p* < 0.05 compared with the CD34^low^-expressing cells. ^#^*p* < 0.05 compared with the parent cells.

Subsequently, CD34^high^ fraction was separated by positive MACS sorting and a CD34^high^-enriched population was obtained (Figure 2(a)). Markers for undifferentiated epithelial cells such as *p63*, *K5*, and *K14* and those for stem cells such as *CD34*, *OCT4*, and *SHH* were consistently expressed at higher mRNA levels and protein levels in CD34^high^-enriched cells than CD34^low^-expressing and parent cells (Figures 2(b) and 2(c)). Holoclone formation was used to indicate potential self-renewal characteristics of stem cell. Freshly isolated CD34^high^-enriched cells formed more holoclones, which are large and round colonies with a smooth boundary mostly containing small cells, than CD34^low^-expressing and parent cells in 2 weeks (Figure 2(d)). Moreover, holoclones formed by CD34^high^-enriched cells appeared larger than those generated by CD34^low^-expressing and parent cells (Figure 2(d)). Holoclones formed in all cell groups could be repeatedly subcultured and generate more holoclones. Though no specific *in vitro* assay can confirm the identity of stem cells, the above evidence supports that CD34^high^-enriched subpopulation isolated from HaCaT cells exhibits stem-like phenotypes.

### 3.3. Increased Resistance of CD34^high^-Enriched Cells to Arsenic Toxicity

**Figure 3:**
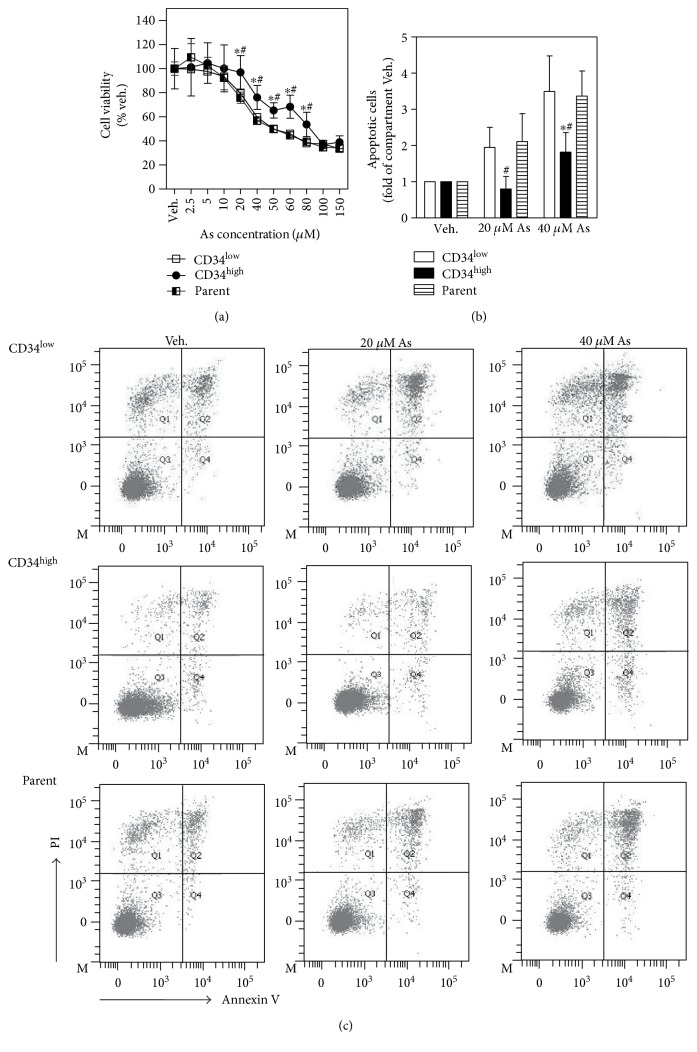
Hyperresistance of CD34^high^-enriched cells to acute arsenic toxicity. (a) Cell viability determined with MTS assay after 24 h exposure to sodium arsenite. *n* = 4. (b) Quantification and (c) representative image for Annexin V-FITC and PI double staining cells determined with flow cytometry. Apoptotic cells after 24 h exposure to sodium arsenite were counted as the cells in both Q2 and Q4. *n* = 3. ^∗^*p* < 0.05 compared with CD34^low^-expressing compartment. ^#^*p* < 0.05 compared with parent compartment.

To test cellular defense against arsenic toxicity, CD34^high^-enriched cells, CD34^low^-expressing cells, and parent cells were acutely (24 h) exposed to sodium arsenite. As shown in Figure 3(a), CD34^high^-enriched cells showed significantly increased viability compared with CD34^low^-expressing and parent cells in response to arsenic from 20 *μ*M to 80 *μ*M. The lethal concentration 50 (LC50) was 88.1 *μ*M for CD34^high^-enriched cells, 58.8 *μ*M for CD34^low^-expressing cells, and 56.7 *μ*M for parent cells. Then, arsenic-induced apoptosis was measured with Annexin V-FITC and PI double staining method. Consistent with the results of cell viability, less apoptotic cells were observed in high-level arsenic-exposed CD34^high^-enriched cells compared with CD34^low^-expressing and parent cells (Figures 3(b) and 3(c)).

### 3.4. High Activity of *NRF2* and ROS Levels in CD34^high^-Enriched Cells

**Figure 4:**
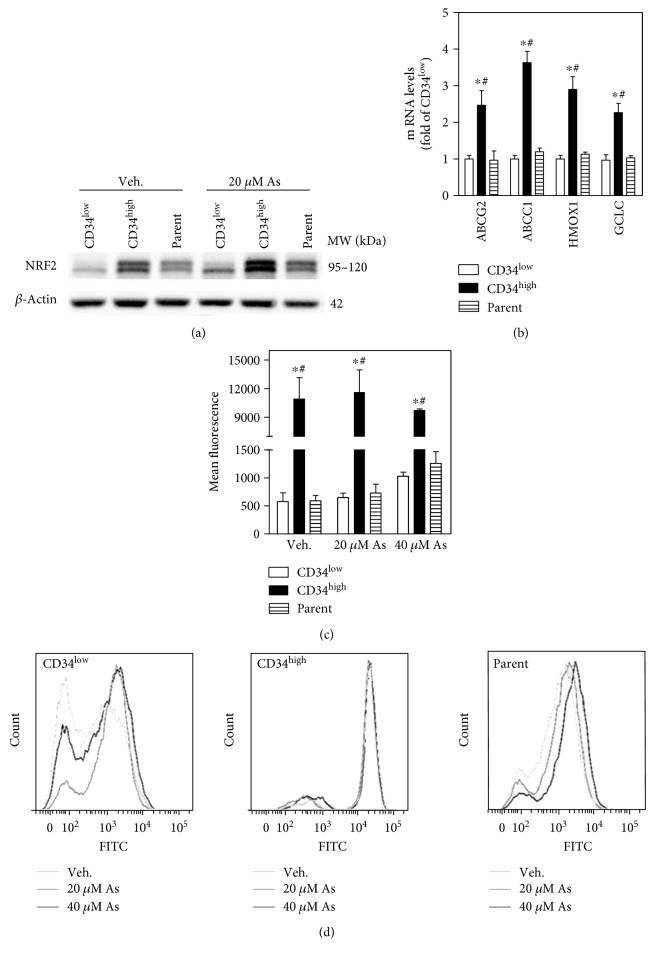
High NRF2 activity and ROS levels in CD34^high^-enriched cells. (a) Representative image of Western blot for NRF2 in CD34^low^-expressing, CD34^high^-enriched, and parent cells. Cells were exposed to Veh. or 20 *μ*M sodium arsenite for 6 h. (b) Innate mRNA levels of NRF2 downstream genes, including those encoding MRPs and antioxidant-related proteins in CD34^low^-expressing, CD34^high^-enriched, and parent cells. (c) Quantification and (d) representative histogram for intracellular ROS determined with flow cytometry. Cells were treated with Veh., 20 *μ*M, or 40 *μ*M sodium arsenite for 2 h. *n* = 3. ^∗^*p* < 0.05 compared with CD34^low^-expressing compartment. ^#^*p* < 0.05 compared with parent compartment.

Western blot showed higher innate and arsenic-induced NRF2 protein levels in CD34^high^-enriched cells than CD34^low^-expressing and parent cells (Figure 4(a)). Consistently, a panel of NRF2 downstream genes, including those encoding multidrug resistance proteins (MRPs) such as ABCG2 and ABCC1, and antioxidant enzymes HMOX1 and GCLC, was increased at the transcript level in CD34^high^-enriched cells compared with CD34^low^-expressing and parent cells (Figure 4(b)). The intracellular ROS levels in CD34^high^-enriched cells were surprisingly higher than CD34^low^-expressing and parent cells under basal conditions (Figures 4(c) and 4(d)). Interestingly, ROS levels were increased to 1.5–2 fold of Veh. in CD34^low^-expressing and parent cells in response to arsenic, but remained stable in CD34^high^-enriched cells (Figures 4(c) and 4(d)).

### 3.5. Increased Export of Arsenic from CD34^high^-Enriched Cells

**Figure 5:**
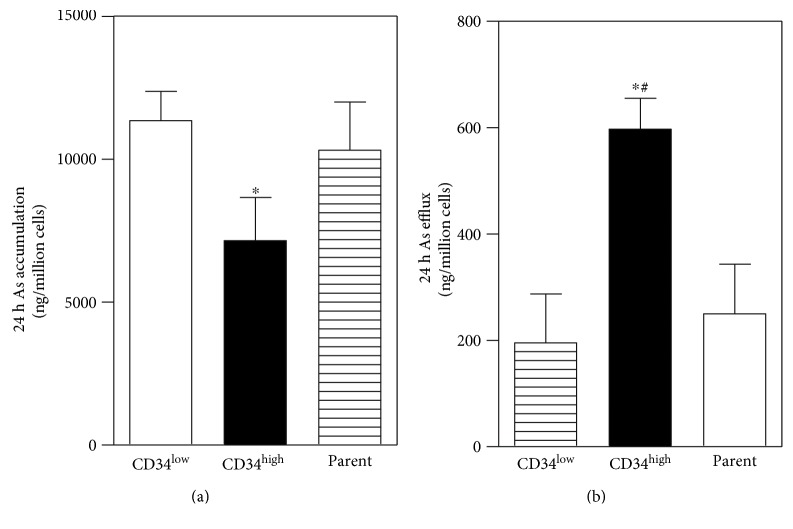
Accumulation and efflux of arsenic in the CD34^low^-expressing, CD34^high^-enriched, and parent cells. (a) Cellular accumulation of arsenic after 24 h exposure to 10 *μ*M of sodium arsenite. (b) 24 h cellular efflux of arsenic into the medium. *n* = 4. ^∗^*p* < 0.05 compared with the CD34^low^-expressing cells. ^#^*p* < 0.05 compared with the parent cells.

Some NRF2 downstream genes, including *MRPs* and *GCLC*, are involved in intracellular arsenic export, and their high expression may contribute to arsenic resistance [35]. Thus, we evaluated arsenic accumulation and export in CD34^high^-enriched cells after acute arsenic exposure. CD34^high^-enriched cells showed significantly lower intracellular arsenic levels than CD34^low^-expressing cells after 24 h arsenic exposure (Figure 5(a)). The reduced intracellular arsenic accumulation may be caused by enhanced arsenic export. As shown in Figure 5(b), after acute exposure to the same level of arsenic, CD34^high^-enriched cells eliminated more accumulated arsenic into the medium in the next 24 h compared with CD34^low^-expressing and parent cells.

### 3.6. Abrogation of Hyperresistance to Arsenic Toxicity in CD34^high^-Enriched Cells due to *NRF2* Silencing

**Figure 6:**
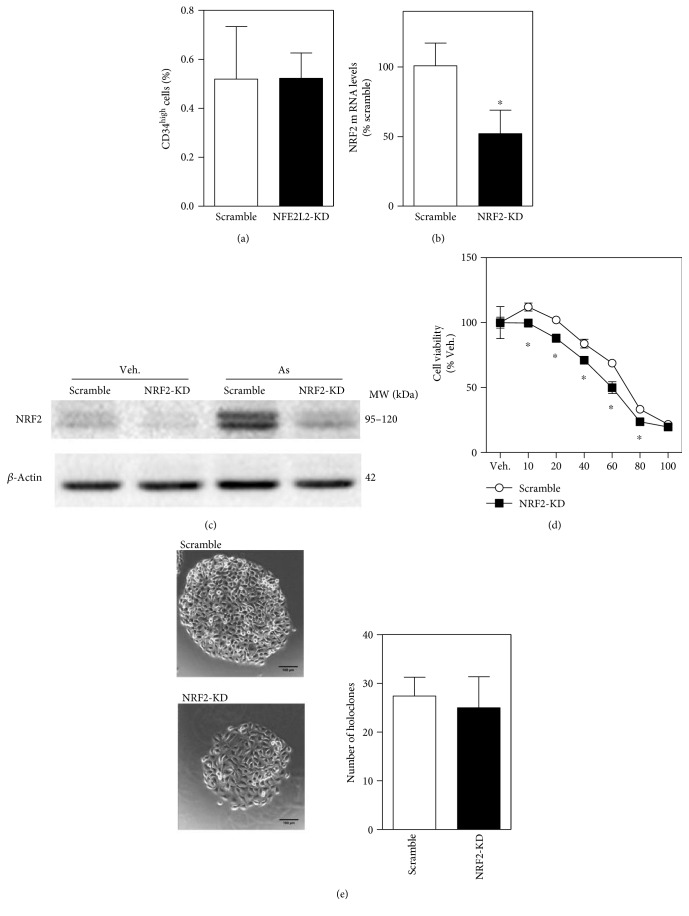
Silencing *NRF2* abolished hyperresistance to acute arsenic cytotoxicity and compromised holoclone formation capacity in CD34^high^-enriched cells. (a) The ratio of CD34^high^ cells in scramble and *NRF2*-KD HaCaT cells. *n* = 3. (b) mRNA levels of *NRF2* determined by RT-qPCR in CD34^high^-enriched cells that were purified from scramble and *NRF2*-KD HaCaT cells. *n* = 3. (c) Representative protein bands of NRF2 determined by Western blot in CD34^high^-enriched cells that were purified from scramble and *NRF2*-KD HaCaT cells. Significantly reduced NRF2 protein level was observed in arsenic-treated (20 *μ*M, 6 h) cells. (d) Cell viability of scramble and *NRF2*-KD CD34^high^-enriched cells determined with MTS assay after 24 h exposure to sodium arsenite. *n* = 4. (e) Holoclones formed by scramble and *NRF2*-KD CD34^high^-enriched cells. Left: representative image of holoclones (400x magnification, bar = 100 *μ*m); right: the average number of holoclones in the 30 mm dish. *n* = 5. ^∗^*p* < 0.05 compared with the scramble compartment.

Since resistance to arsenic toxicity and increased cellular arsenic export of CD34^high^-enriched cells can be attributed to high NRF2 activity, we subsequently examined what if *NRF2* was silenced. *NRF2*-KD did not change the proportion of CD34^high^ cells in HaCaT cells (Figure 6(a)), suggesting that reduced *NRF2* expression does not affect expression of CD34. CD34^high^-enriched cells isolated from *NRF2*-KD cells showed significant decreases in *NRF2* mRNA levels (Figure 6(b)), which were approximately 50% of scramble. Western blot showed that NRF2 protein expression was obviously reduced in *NRF2*-KD cells than scramble cells, especially when cells were treated with 20 *μ*M sodium arsenite (Figure 6(c)). Decreased cell viability in *NRF2*-KD CD34^high^-enriched cells compared with scramble compartments in response to 10 *μ*M to 80 *μ*M arsenic, ranging from 9% to 29%, was observed. (Figure 6(d)). In addition, *NRF2*-KD in CD34^high^-enriched cells did not affect the number of holoclones but reduced the size of holoclones compared with scramble (Figure 6(e)). Meanwhile, cells in *NRF2*-KD holoclones were not as small or as compact as in scramble (Figure 6(e)).

## 4. Discussion

In the present study, we found that CD34^high^-enriched cells purified from the HaCaT cell line exhibited a stem-like phenotype. In addition, the CD34^high^ stem-like cells showed hyperresistance to arsenic toxicity compared with the mature cells. This hyperresistance was associated with higher innate expression of *NRF2* and its downstream genes in CD34^high^ cells. These data support the hypothesis that skin stem/progenitor cells have a survival selection advantage in response to arsenic exposure, which serves as a possible mechanism for the observed overabundance of stem cells during arsenic carcinogenesis [7–9]. Our results are consistent with the previous report by Tokar et al. [27], which found that the WPE human prostate stem cells showed innate resistance and hyperadaptability to arsenic with higher transcript expression of *NRF2*, *ABCC1*, and *GST-pi* than the parent RWPE-1 cells.

Although the role of stem cells in arsenic adaptation is still poorly understood, stem cells are usually resistant to chemical toxicity and act as a “strategic” reserve for wound repair from chemical insult [26, 37]. Resistance to toxins of stem cells is mostly observed in cancer stem cells and is largely attributed to overexpression of antioxidant proteins and low intracellular levels of reactive oxygen species. As a master transcription factor for antioxidant proteins, NRF2 is likely to account for the toxic-resistance feature of normal stem cells and cancer stem cells [38]. With respect to normal stem cells, exogenous NRF2 transduction or pharmacological NRF2 activation (e.g., tBHQ or melatonin) prevented toxin-induced cell death in human or mouse neural stem cells [30, 39, 40]. In rat cardiac stem cells, NRF2 upregulation induced by resveratrol was found to enhance cell survival and improve cardiac function [41]. In murine mesenchymal stem cells, NRF2 upregulation reduced oxidative stress-induced apoptosis and cytotoxicity [42]. A normal human prostate stem cell line, the WPE-stem cell, showed innate resistance to arsenic cytotoxicity and high transcript expression of antiapopototic, antistress, and drug adaption genes including *NRF2* [27]. As for cancer stem cells, higher expression or activity of NRF2 has been demonstrated in glioma, lung, esophageal, breast ovarian, and colon stem cells [43–46]. Our study provides the direct evidence that skin stem/progenitor cells preserve high NRF2 activity. The difference in cell viability between scramble and *NRF2*-KD CD34^high^-enriched cells ranged from 9% to 29%, which is relatively mild and indicates that other mechanisms independent of NRF2 are involved in cell resistance to arsenic. However, this difference is close to what we have observed in comparison of cell viability between CD34^low^-expressing and CD34^high^-enriched cells. Thus, the relatively high NRF2 expression/activity may play a key role in hyperresistance to arsenic of skin stem/progenitor cells than their mature compartments.

NRF2 is considered to facilitate chemical resistance through several mechanisms: (1) upregulating downstream antioxidants and detoxifying enzymes such as GCLC, GCLM, and HOMX1 [47]; (2) inducing MRPs that export chemicals from the cell [18–20]; (3) interacting with antiapoptotic pathway resulting in attenuation of chemical-induced apoptosis [48]; and (4) enhancing proteasome activity leading to activation of the survival-related pathway [49]. In the present study, a panel of NRF2 downstream genes was highly expressed in CD34^high^-enriched cells. High transcript levels of *GCLC* indicate a potential enhanced glutathione (GSH) synthesis. In addition to antioxidant property, GSH mitigates arsenic toxicity by increasing arsenic export through formation of arsenic-GSH complexes [50]. These complexes can be formed either nonenzymatically [51] or enzymatically by GST-pi [52]. GSH also affects arsenic resistance by participating in arsenic methylation through reduction of pentavalent arsenic species to trivalent ones [53]. However, HaCaT cells are arsenic-methylation deficient. MRPs are export pumps in xenobiotic metabolism and showed higher transcript levels in CD34^high^-enriched cells. All are consistent with the observed low accumulation but high export of arsenic in CD34^high^-enriched cells. Therefore, in addition to the well-known antioxidant activation, elevated arsenic export associated with high NRF2 activity may be involved in hyperresistance to arsenic in CD34^high^ stem-like cells.

NRF2 also plays an important role in stem cell biology; however, the reports are controversial. The homolog of NRF2 in *Drosophila*, CncC, was continuously active in intestinal stem cells of *Drosophila* and maintained quiescence of stem cells [54]. CncC inhibition mediated by KEAP1 was required for proliferation of these stem cells [54]. In human embryonic stem cells (hESCs), *NRF2* was highly expressed compared with differentiated cells, while a reduction of NRF2 activity induced cell differentiation and inhibited pluripotency of the stem cells [28]. In human airway basal stem cells, NRF2 activation promoted cell proliferation by regulating NOTCH1 and antioxidant program [55]. In skeletal muscle stem/progenitor cells, NRF2 supported cell proliferation by transcriptional upregulating *MyoD* and facilitated muscle regeneration [29]. Increased NRF2 activity enhanced both proliferation and differentiation of neuronal progenitor cells [30]. In hematopoietic stem cells (HSCs), NRF2 activation was required for maintenance of quiescence [56, 57]. Meanwhile, NRF2 activation determined differentiation fate of HSCs, driving the myeloid differentiation but suppressing erythoid or lymphoid differentiation [58]. Though most studies support that NRF2 is involved in pluripotency, proliferation, and differentiation of stem cells, it appears that the exact role of NRF2 in homeostasis of stem/progenitor cells is tissue and cell-type specific. In the present study, *NRF2* silencing does not affect population size of stem/progenitor cells under basal conditions. However, *NRF2*-KD CD34^high^-enriched cells formed smaller holoclones compared with scramble compartments, indicative of the role of NRF2 in self-renewal in these cells. NRF2 activity is suggested to be closely associated with cell cycle regulation. Cell cycle was arrested in G1 phase in *NRF2*-knockdown human breast cells with mitoquinone treatment [59]. While G2/M arrest resulted from *NRF2* deficiency was observed in mouse alveolar epithelia [60] and human glioma stem cells [61]. Therefore, our data are consistent with previous findings and provide new information on the role of NRF2 in skin stem/progenitor cells.

Stem cells undergoing self-renewal are indicated to possess low intracellular ROS levels, while elevated ROS levels switch stem cell self-renewal to differentiation [62]. As a potent regulator of stem cell self-renewal and differentiation, NRF2 is suggested to function at least in part due to its antioxidant capacity. However, the functions of NRF2 through regulating ROS in stem cells are diversely reported and presumably context dependent. In some cases, NRF2 may regulate proliferation of stem cells independent upon ROS [56], but directly bind to ARE in promoter region of some stem cell self-renewal genes, such as *CXCR4* [57] and *NOTCH1* [55]. It is quite interesting to observe that CD34^high^-enriched cells presented with higher NRF2 expression/activity, as well as markedly elevated intracellular ROS levels, than mature compartments under basal conditions. This unexpected high intracellular ROS levels in the putative skin stem-like cells may suggest diverse redox balance in different cell/tissue types and even in cells of the same origin with different differentiating stages. However, the underlying mechanisms cannot be clearly clarified in the present study. Arsenic exposure is well-known to induce enhanced NRF2 protein levels as we have seen in the present study (Figure 4(a)). This NRF2 induction was accompanied with increased ROS levels in the mature compartments (CD34^low^-expressing and parent cells), but without alteration of ROS levels in CD34^high^-enriched cells (Figures 4(c) and 4(d)). One possible explanation for this phenomenon is that innate high expression of NRF2 and antioxidant proteins in CD34^high^-enriched cells veiled the alteration of ROS at the determination time point. Also, arsenic can activate NRF2 through other mechanisms such as NRF2 stabilization by p62-mediated sequestration of KEAP1 resulting from autophagy inhibition [63].

## Supplementary Material

Table S1. Genes and primers for RT-qPCR.
