# Supplementary material for: NRF2 Is a Potential Modulator of Hyperresistance to Arsenic Toxicity in Stem-Like Keratinocytes
Source: Oxid Med Cell Longev. 2017 Sep 10;2017:7417694. doi: 10.1155/2017/7417694 (PMC5610874; doi:10.1155/2017/7417694)
Supplement: Supplementary file 1 — Table S1. Genes and primers for RT-qPCR. [file 7417694.f1.doc]

**Supplementary materials**

Table S1. Genes and primers for RT-qPCR

| Gene | GenBank Accession No. | Primers (5’→3’) |
| --- | --- | --- |
| *p63* | NM_003722.4, NM_001114978.1, NM_001114980.1,  NM_001114981.1, NM_001329148.1, NM_001329145.1, NM_001329146.1, NM_001329149.1, NM_001329150.1, NM_001329144.1 | Forward: CCCCAAGCAGTGCCTCTACA  Reverse: CGTGAATCGCACAGCATCAA |
| *CD34* | NM_001773.2, NM_001025109.1 | Forward: TTCACTGAGCAAGATGTTGCAA  Reverse: ACCAGTGCAATCAGGGTCTTTT |
| *OCT4* | NM_001285987.1, NM_002701.5, NM_001173531.2, NM_001285986.1, NM_203289.5 | Forward: GAAACCCACACTGCAGCAGAT  Reverse: GAACCACACTCGGACCACATC |
| *K5* | NM_000424 | Forward: GTAGCAGCTCCAGCGTCAAAT  Reverse: TTGGAAGGCAGTGACTTGCA |
| *K14* | NM_000526 | Forward: ACCTCTCCTCCTCCCAGTTCTC  Reverse: TGCACATCCATGACCTTGGT |
| *SHH* | NM_000193 | Forward: GGAGCGGACAGGCTGATG  Reverse: ACTCCTGGCCACTGGTTCAT |
| *ABCG2* | NM_004827.2, NM_001257386.1 | Forward: AGACTTTAAAGCCACAGAGATCA  Reverse: TCACCCCCGGAAAGTTGATG |
| *ABCC1* | NM_004996.3 | Forward: CCCGCTCTGGGACTGGAA  Reverse: GTAGAAGGGGAAACAGGCCC |
| *RPS18* | NM_022551.2 | Forward: TAGCCAATGGTCTGGACAACA  Reverse: CCTCTATGGGCCCGAATCTT |
| *HMOX1* | NM_002133.2 | Forward: GAGCACAGAGCCTCGCCTTT  Reverse: TCATCATCCATGGTGAGCTGG |
| *GCLC* | NM_001498 | Forward: GATGCTGTCTTGCAGGGAATG  Reverse: AGCGAGCTCCGTGCTGTT |
| *β-ACTIN* | NM_001101.3 | Forward: GAGCACAGAGCCTCGCCTTT  Reverse: TCATCATCCATGGTGAGCTGG |
| *NRF2* | NM_006164.4, NM_001145412.3, NM_001145413.3, NM_001313900.1, NM_001313901.1 | Forward: AGCCCAGCACATCCAGTCA  Reverse: TGTGGGCAACCTGGGAGTAG |

All primers are from Sigma-Aldrich.
